# FASconCAT-G: extensive functions for multiple sequence alignment preparations concerning phylogenetic studies

**DOI:** 10.1186/s12983-014-0081-x

**Published:** 2014-11-18

**Authors:** Patrick Kück, Gary C Longo

**Affiliations:** Zoologisches Forschungsmuseum A. Koenig, Adenauerallee 160-163, Bonn, 53113 Germany; Center for Ocean Health, 100 Shaffer Road, Santa Cruz, 95060 CA USA

**Keywords:** Multiple sequence alignment, Phylogenetic reconstruction, Sequence processing, Consensus sequence, Sequence translation, Sequence concatenation, File format conversion

## Abstract

**Background:**

Phylogenetic and population genetic studies often deal with multiple sequence alignments that require manipulation or processing steps such as sequence concatenation, sequence renaming, sequence translation or consensus sequence generation. In recent years phylogenetic data sets have expanded from single genes to genome wide markers comprising hundreds to thousands of loci. Processing of these large phylogenomic data sets is impracticable without using automated process pipelines. Currently no stand-alone or pipeline compatible program exists that offers a broad range of manipulation and processing steps for multiple sequence alignments in a single process run.

**Results:**

Here we present FASconCAT-G, a system independent editor, which offers various processing options for multiple sequence alignments. The software provides a wide range of possibilities to edit and concatenate multiple nucleotide, amino acid, and structure sequence alignment files for phylogenetic and population genetic purposes. The main options include sequence renaming, file format conversion, sequence translation between nucleotide and amino acid states, consensus generation of specific sequence blocks, sequence concatenation, model selection of amino acid replacement with ProtTest, two types of RY coding as well as site exclusions and extraction of parsimony informative sites. Convieniently, most options can be invoked in combination and performed during a single process run. Additionally, FASconCAT-G prints useful information regarding alignment characteristics and editing processes such as base compositions of single in- and outfiles, sequence areas in a concatenated supermatrix, as well as paired stem and loop regions in secondary structure sequence strings.

**Conclusions:**

FASconCAT-G is a command-line driven Perl program that delivers computationally fast and user-friendly processing of multiple sequence alignments for phylogenetic and population genetic applications and is well suited for incorporation into analysis pipelines.

**Electronic supplementary material:**

The online version of this article (doi:10.1186/s12983-014-0081-x) contains supplementary material, which is available to authorized users.

## Introduction

Phylogenetic and population genetic analyses commonly involve the manipulation and processing of multiple sequence alignments. For instance, concatenation of multiple gene alignments are common in rRNA analyses (e.g. [1-6]) and in ’mixed’ nucleotide alignment analyses, combining rRNA genes like 18S and 28S as well as protein coding nucleotide sequences (e.g. [7-10]). Likewise, the ability to concatenate hundreds to thousands of nucleotide or amino acid single gene alignments has recently become an indispensable tool with the growth of phylogenomics (e.g. [11-24]). Sequence translation of nucleotide data (DNA/RNA) to protein coding sequences as well as RY coding [25] of nucleotide sequences are common practices to reduce the signal-to-noise ratio of underlying data in phylogenomic studies prior to tree reconstruction (e.g. [26-29]). In order to predict possible nucleotide sequences for a specified protein, researchers often reverse translate amino acid sequences to nucleotide states (e.g. [30-33]). Another common analysis of multiple sequence alignments is consensus sequence generation, which is commonly used to identify and compare conserved and variable regions (e.g. [34-36]), design degenerated PCR primers for appropriate locations within the alignment, or to define operational taxonomic units using DNA barcode data for subsequent phylogenetic analysis (e.g. [37]). Consensus sequence generation has also become a valuable tool in large scale population genetic analyses that pool individuals as a cost effective method for determining population level data. Recent studies searching for genes potentially under selection among populations relied on identifying the most common allele at polymorphic sites as well as alleles fixed within populations [38], which can be accomplished through consensus generation.

Phylogenetic and population genetic analyses also commonly involve the tedious tasks of dealing with different sequence file formats and sequence renaming with the later becoming increasingly time-consuming when dealing with hundreds of sequences.

Although there are many scripts and online platforms that address these issues or manipulate sequence alignments with single processing steps, a software tool which enables combined processing steps in a single operation is lacking. Software like SequenceMatrix [39], TranslatorX [40], and CONCATENATOR [41] are pure concatenation tools which can be used only via graphical user interface or which are web server designed and therefore cannot be implemented in automatic process pipelines. 2matrix [42] is a pure concatenation tool as well but command line driven. SCaFoS [43] is a phylogenomic tool for selecting and concatenating sequences in large multigene and species datasets at either the amino acid or nucleotide level. Although SCaFoS is efficient at selecting orthologous sequences, creating chimerical sequences, and selecting genes according to their level of missing data, it lacks alignment processing options such as sequence translation, RY-coding, secondary structure handling, sequence renaming and consensus sequence generation.

With FASconCAT-G (FcC-G), we introduce a versatile software designed for processing and manipulating multiple sequence alignments. Conveniently, FcC-G allows for multiple processing steps in a single run and is easily implemented into pipeline analyses. FcC-G represents an advancement of FASconCAT [44], an already commonly used tool in phylogenetic studies (e.g. [45-53]).

## Results and discussion

**Figure 1:**
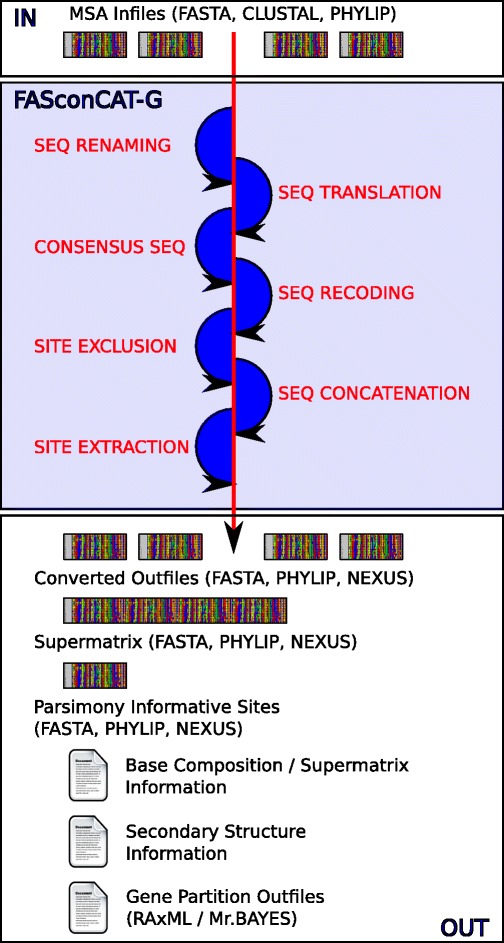
**Simplified flowchart depicting FcC-G implemented options.** Possible alignment input file formats, which can be processed individually or in combination, are listed in the top section. The hierarchical flow of possible processing options is depicted by the direction of the arrow in the middle of the figure. Processing options can be combined following the flow direction. For processing chains that contradict this flow direction, FcC-G has to be run twice. Possible output files of FcC-G are depicted on the bottom section. The content of output files depends on the chosen processing options and the type of sequences in given input file alignments. A more detailed description of possible FcC-G input/output files, implemented processing options, and examples of complex process chains are discussed in the FcC-G manual.

FASconCAT-G accepts multiple nucleotide, amino acid, and structure sequence alignment input files and can perform sequence renaming, file format conversion, sequence translation of nucleotide and amino acid states, consensus generation of specific sequence blocks, sequence concatenation, RY coding, model selection of amino acid replacement using ProtTest [54], extraction of parsimony informative sites as well as generation of partitioned files for MrBayes [55] and RAxML analyses [56]. The process order of FcC-G allows for a wide range of optional process combinations (Figure 1), although, some process chains are not possible in a single process run. For instance, it is not possible to RY code nucleotide sequences before translating them to amino acid sequences or to build consensus sequences before the sequence translation process. For tasks of this nature, FcC-G has to be run twice. However, we hope the current process order of FcC-G is useful for most phylogenetic and population genetic applications. To avoid errors such as the exclusion of third nucleotide site positions before sequence translation to amino acid character states, FcC-G contains a hierarchical order of single file processing steps:

1. Sequence renaming
2. Sequence translation (nucleotide to amino acid sequences or vice versa)
3. Generation of consensus sequences of predefined sequence blocks
4. RY coding of nucleotide sequences
5. Exclusion of each third nucleotide site position
6. Sequence concatenation
7. Extraction of parsimony informative sites
8. Print out of edited sequences and additional sequence information

### Sequence renaming

Sequence names are often coded during the sequencing process or, if downloaded from NCBI, extended with additional information and non-alphanumeric signs which are often not allowed in downstream analysis programs. Accordingly, FcC-G can rename defined sequence names prior to file processing by using a user supplied info file, which lists, in each row, the old name delimited from the new name by a tabstop. Sequences which are not listed in the user supplied info file remain unchanged. FcC-G will print additional information of the sequence renaming process to a new outfile.

### Sequence translation

FcC-G can translate standard nucleotide sequence states to amino acid characters and vice versa. For sequence translation of nucleotide data FcC-G uses the standard IUPAC triplet codes for amino acid characters. When translating amino acid states to corresponding nucleotide characters, FcC-G uses compressed IUPAC codes. Conveniently, FcC-G can recognize and handle both amino acid and nucleotide data sets in a single processing run. Accordingly, FcC-G will only translate sequences of infiles which are suitable for a defined translation process. This makes it very easy to concatenate a mixture of different infile sequence types to one specific supermatrix sequence type FcC-G will translate incomplete nucleotide triplets to ‘?’. FcC-G translates nucleotide triplets even if triplets contain ambiguity codes, provided that the triplets are still assignable to specific amino acid characters (e.g. ‘YTR’ ↪ Leucine/L). Otherwise, unspecific triplets are translated to ‘?’ (e.g. ‘RCT’ ↪ ?). FcC-G does not check for correctness of given reading frames but will print a warning in the terminal window if sequence lengths are not a multiple of three.

### Consensus sequences

FcC-G can create consensus sequences for matching defined sequence blocks within given infiles using one of three consensus methods: ‘Most Frequent Consensus’, ‘Majority Rule Consensus’, and ‘Strict Consensus’. The ‘Most Frequent Consensus’ option considers the most frequent character state at a given site among defined sequence blocks as the consensus character state. If two or more character states are equally frequent, FcC-G uses either the corresponding IUPAC ambiguity code as the consensus character state (nucleotide data) or a ‘?’ (amino acid data and nucleotide data). The ‘Majority Rule Consensus’ option considers character states which occur at a given site position in more than 50% of sequences of a defined sequence block as consensus character state. Otherwise, FcC-G uses a ‘?’ as the consensus character state (amino acid data and nucleotide data). The ‘Strict Consensus’ option considers all character states at a given site position to generate a strict consensus sequence for a defined sequence block using IUPAC ambiguity codes for nucleotide data and a ‘X’ for amino acid data. For nucleotide data, indel events (coded as ‘-’) and missing data (coded as ‘?’) are ignored using ‘Strict Consensus’ as long as a nucleotide character state exists for a specific site position. If a specific site on a defined sequence block consists of only indel events (‘-’) and missing data states (‘?’), FcC-G will output a ‘?’ as the consensus character state.

### RY coding of nucleotide sequences

RY coding can be applied to each third nucleotide sequence position or to complete nucleotide sequences. The R code is used for purine states while the Y code is used for pyrimidines. Amino acid sequences are left unchanged unless the sequence translation option from amino acid to nucleotide states has been defined.

### Sequence concatenation

FcC-G can concatenate sequence alignment infiles (nucleotide and amino acid as well as ‘dot-bracket’ structure information) of identical taxa into a supermatrix file. It is also possible to concatenate amino acid and nucleotide alignments into one supermatrix. In the supermatrix file, taxon sequences which were missing from single files are encoded either by ‘N’ (nucleotide sequences), ‘X’ (amino acid sequences) or by ‘. ’ (dots structure strings in ‘dot-bracket’ format).

### Extraction of parsimony informative sites

FcC-G can print out additional information file(s) identifying parsimony-informative sites of given infiles and/or the concatenated supermatrix. A site is parsimony-informative if it contains at least two types of nucleotides (or amino acids), and at least two of them occur with a minimum frequency of two. The file format of parsimony-informative alignment files depends on the chosen output format(s).

### Input/Output

FcC-G can simultaneously handle three different infile formats (FASTA, CLUSTAL, and PHYLIP) in any combination. Similarly, FcC-G can print concatenated and/or edited alignment files in FASTA, NEXUS, and/or PHYLIP format but FASTA is the default. NEXUS outfiles can conveniently be imbedded with MrBayes commands for direct execution in PAUP [57] or MrBayes [58] (very convenient for partitioned or mixed DNA/RNA analyses) or output without any specific commands. Likewise, PHYLIP output files can be directly used for Maximum Likelihood tree reconstruction analyses with RAxML [56] or PhyML [59]. Additionaly, our new software tool prints a file with useful information about alignment and sequence characteristics for the concatenated supermatrix as well as all single infiles. Information on this file includes single base compositions (including GC content), sequence types as well as sequence lengths and the number of taxa represented in each infile and the concatenated supermatrix. The file also contains information specific to the concatenation process, such as the position of each sequence fragment in the concatenated supermatrix as well as a list of all concatenated sequences and inserted replacement strings. However, the evaluation of this additional information often results in longer computation times depending on the size of data sets. Therefore, FcC-G offers an option to increase the overall computation speed by decreasing the information sampled and printed to the information file. If one or more infiles contain a secondary structure string, FcC-G will print another file with information about stem and loop character states and positions in both the concatenated supermatrix and infile(s). FcC-G can also print parsimony informative sites (sites which consist of at least two types of nucleotides, or amino acids, with a minimum frequency of two) from given infiles and/or the concatenated supermatrix to separate output files. Furthermore, FcC-G can optionally generate additional gene partition output files for the concatenated supermatrix which can be directly used for Maximum Likelihood analyses using RAxML [56] or for Bayesian analyses with MrBayes [58].

### Model selection of amino acid replacement using ProtTest

FcC-G offers the option to generate the best-fit protein model for each amino acid gene partition in RAxML partition formatted supermatrices using the external software, ProtTest [54]. The ProtTest option can only be executed with amino acid infiles or translated infiles and when sequence concatenation has been chosen together with the partition option (“-l”), but not for supermatrices in NEXUS format. FcC-G implements the default parameters for ProtTest version 3.3 and uses the ProtTest BIC criterium to select the best-fit model.

## Conclusions

**Figure 2:**
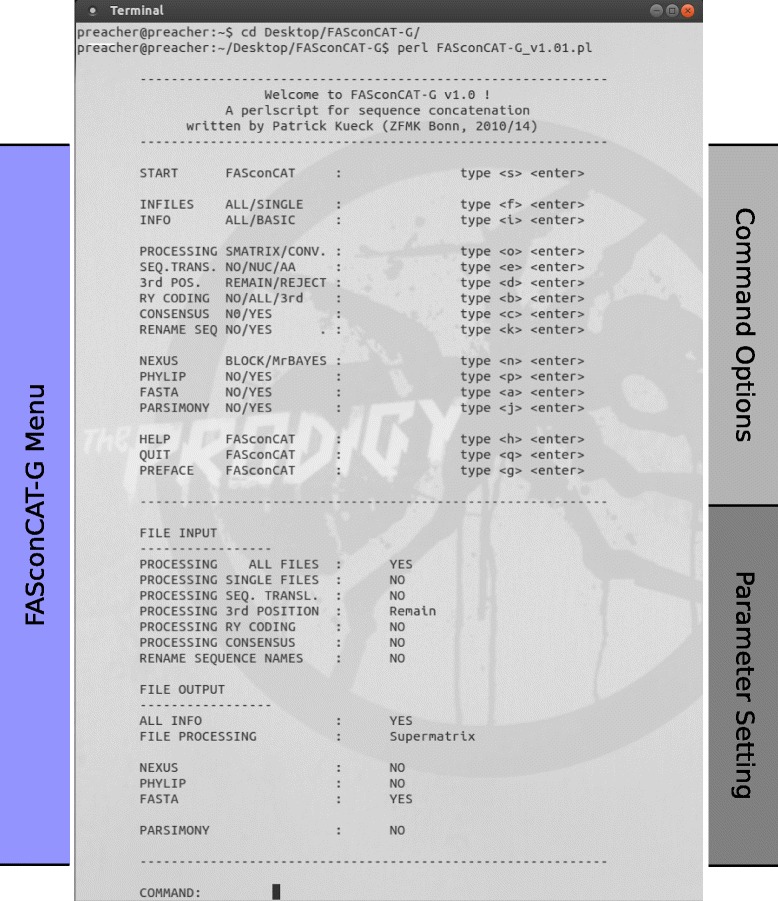
**Terminal menu of FcC-G.** The menu is subdivided into a command block (upper half) and a setting block (lower half). Users can specify their setting by using single commands via menu options or by typing multiple commands directly via the start command line of FcC-G.

With FcC-G, we introduce an advanced editor to facilitate subsequent processing steps for multiple sequence alignments in phylogenetic and population genetic studies. Like its predecessor version, FASconCAT, FcC-G is easy to use, very fast (even with large data sets) and not limited in number of input files or input sequences. It facilitates data handling, it is time saving in generating and processing data matrices, and provides useful additional information about input sequences. FcC-G is implemented in Perl and runs on Windows PCs, Mac OS and Linux operating systems. FcC-G is command-line driven and well suited for incorporation into automatic process pipelines. Alternatively, the software tool can be operated and executed through interactive terminal menu options (Figure 2). Most processing options of FcC-G are combinable (Figure 1) and help is provided for every option. The executable source code (Additional file 1) as well as example test files and a detailed documentation of FcC-G are freely available at https://www.zfmk.de/en/research/research-centres-and-groups/fasconcat-g. The program is open-source and released under the terms of the GNU General Public License (GPL) 3.0. Detailed information and instructions are provided in the manual of FcC-G (Additional file 2). The manual also includes some practical examples, which demonstrate FcC-G is a suitable and user-friendly tool for complex phylogenetic and population genetic data processing.

## Methods

FcC-G is implemented in Perl (Perl 5.0 or higher) and platform independent. Like the predecessor version FASconCAT, FcC-G can be used via command line or by terminal menu options. The terminal menu is subdivided into two parts, separated by a dashed line (Figure 2). The upper component constitutes of a list of all possible options and their associated commands for adjustment. The lower part shows the actual parameter settings of FcC-G. All default parameters can be optionally changed, and the new setting configuration will be displayed in the lower part of the menu. FcC-G is distributed under GNU GPL 3.0 and freely available from https://www.zfmk.de/en/research/research-centres-and-groups/fasconcat-g or upon request from the corresponding authors.

## Additional files

Additional file 1
**Executable Perl script of FASconCAT-G.** FASconCAT-G is distributed under GNU GPL 3.0 and freely available. Windows users have to install a PERL interpreter on their operating system. Mac and Linux users can directly start FASconCAT-G via terminal options.

Additional file 2
**FASconCAT-G manual.** Detailed information and instructions and practical examples of FASconCAT-G. The pdf document can be opened with pdf readers like AdobeAcrobatReader, Xpdf, or DocumentViewer.
