# Supplementary material for: FASconCAT-G: extensive functions for multiple sequence alignment preparations concerning phylogenetic studies
Source: Front Zool. 2014 Nov 18;11:81. doi: 10.1186/s12983-014-0081-x (PMC4243772; doi:10.1186/s12983-014-0081-x)
Supplement: Additional file 2 — FASconCAT-G manual. Detailed information and instructions and practical examples of FASconCAT-G. The pdf document can be opened with pdf readers like AdobeAcrobatReader, Xpdf, or DocumentViewer. [file 12983_2014_81_MOESM2_ESM.pdf]

Patrick Kück & Gary C. Longo

FASconCAT-G

June 2014

Contents

1 Introduction **3**

2 Usage/Options **3**
2.1 Start FASconCAT-G via menu 4
2.1.1 Open the menu under Windows 4
2.1.2 Open the menu under Linux/Mac 4
2.1.3 Menu handling 4
2.2 Start FASconCAT-G via single command line 5
2.3 Options 6
2.3.1 File Processing (-o option) 6
2.3.2 Sequence Translation (-e option) 6
2.3.3 Renaming Sequence Names (-k option) 7
2.3.4 Rejection of 3rd Nucleotide Codon Positions (-d option) 8
2.3.5 RY Coding (-b option) 8
2.3.6 Create Consensus Sequences (-c option) 9
'Most Frequent Consensus'. 10
'Majority Rule Consensus'. 10
'Strict Consensus'. 11
2.3.7 Handling of Defined Input Files (-f option) 11
2.3.8 Print OUT of Parsimony Informative Sites (-j option) 12
2.3.9 Print OUT Partition File(s) (-l option) 12
2.3.10 Using ProtTest for Amino Acid Model Specification (-m option) 13
2.3.11 Switch Off Print OUT File(s) in FASTA format (-a option) 14
2.3.12 Print OUT File(s) in NEXUS format (-n option) 14
2.3.13 Print OUT File(s) in PHYLIP format (-p option) 15
2.3.14 Reduction of Information Print OUT (-i option) 16

3 Internals **17**
3.1 Input/Output 17
3.2 Secondary Structure Information 19
3.3 Hierarchical Order of File Processing Options 19
3.4 License/Help-Desk/Citation 20

4 Example Usage **21**
4.1 Example 1 21
4.2 Example 2 22
4.3 Example 3 23

5 Copyright **23**

List of Figures

1 FASconCAT-G Menu . . . 4
2 Example Usage 1 of File Processing . . . 6
3 Example Usage 2 of File Processing . . . 6
4 Example Usage 3 of File Processing . . . 6
5 Example Usage 1 of Sequence Translation . . . 7
6 Example Usage 2 of Sequence Translation . . . 7
7 Example Usage 1 of 3rd Nucleotide Codon Position Exclusions . . . 8
8 Example Usage 2 of 3rd Nucleotide Codon Position Exclusions . . . 8
9 Example Usage 1 of Consensus Sequence Option . . . 9
10 Example Usage 2 of Consensus Sequence Option . . . 9
11 Example Usage of Defined Infile Option . . . 12
12 Example Usage 1 of Parsimony Informative Site Print OUT . . . 12
13 Example Usage 2 of Parsimony Informative Site Print OUT . . . 12
14 Example Usage of Non-FASTA Formated Print OUT . . . 14
15 Example Usage 1 of NEXUS (Block) Formated Print OUT . . . 14
16 Example Usage 2 of NEXUS (MrBAYES) Formated Print OUT . . . 14
17 Example Usage 1 of PHYLIP (Strict) Formatted Print OUT . . . 15
18 Example Usage 2 of PHYLIP (Relaxed) Formatted Print OUT . . . 16
19 Example Usage 1 of Combined FASconCAT-G Options . . . 21
20 Example Usage 2 of Combined FASconCAT-G Options . . . 22
21 Example Usage 3 of Combined FASconCAT-G Options . . . 23

List of Tables

1 Option codes for single command line start . . . 5
2 Example Format of User Defined Infile for Sequence Renaming . . . 7
3 Example of Defined Consensus Sequence Blocks . . . 9
4 Example of a Most Frequent Consensus Block . . . 10
5 Example of a Majority Rule Consensus Block . . . 10
6 Example of a Strict Consensus Sequence Block . . . 11
7 File selection menu . . . 11
8 MrBayes Set Up for NEXUS Partition File Output . . . 13
9 MrBayes set up for NEXUS output . . . 15
10 List of Default Information Content . . . 16
11 List of Reduced Information Content . . . 17
12 Overview of possible input and output formats . . . 17
13 Known FASTA Infile Formats . . . 18
14 CLUSTAL format . . . 18
15 Known PHYLIP Infile Formats . . . 19
16 Additional Structure String Information . . . 19

1 Introduction

FASconCAT-G offers a wide range of possibilities to edit and concatenate multiple nucleotide, amino acid, and structure sequence alignment files for phylogenetic and population genetic purposes. The main options include sequence renaming, file format conversion, sequence translation, consensus generation of predefined sequence blocks, and RY coding as well as site exclusions in nucleotide sequences. FASconCAT-G implemented process options can be invoked in any combination and performed during a single process run. FASconCAT-G can also read in and handle different file formats (FASTA, CLUSTAL, and PHYLIP) in a single run. The program is designed to handle multiple sequence alignments, however, sequences must have equal length within each file. FASconCAT-G extracts taxon specific associated gene or structure sequences out of given input files and links them to one string. Missing taxon sequences in single files are replaced either by 'N', 'X' or by '.' (dots) depending on their taxon associated data type (nucleotide, amino acid or "dot-bracket" structures). It is possible to concatenate nucleotide and amino acid files to one supermatrix file. FASconCAT-G can read sequences in interleaved and non-interleaved format.
FASconCAT-G was written on Linux and runs on Windows PCs, Mac OS and Linux operating systems. The program can be started directly via command line or through interactive menu options. Input files coming from Windows CRLF line feeds should be converted into Unix (LF) line feeds. This can be done in several editors (e.g., Bioedit or Notepad++). FASconCAT-G usually recognizes and converts these files but not in every instance.
Ambiguities, indels ('-'), and missing characters ('?'), are allowed, however, any other character in sequences except for those covered by the universal DNA/RNA or amino acid code will lead to an unacceptable error prompt. Structure information in ribosomal sequences is also recognized, analyzed and concatenated by FASconCAT-G if present once in each file and associated with matching sample names. Otherwise, FASconCAT-G will interrupt with a specific error prompt. Besides the concatenation and sequence conversion processes, FASconCAT-G delivers additional sequence information about each input file and the new concatenated supermatrix. The extent of information depends on the chosen information output setting.
The default information output file ("FcC_info.xls") includes information about concatenated areas of each sequence fragment in the concatenated supermatrix, a list of all concatenated sequences, and several other sequence characteristics. In addition to FASTA format, FASconCAT-G can also output concatenated and/or converted sequence files in NEXUS or PHYLIP format. NEXUS outfiles can be imbedded with MrBayes commands for direct execution in PAUP or output without any specific commands. PHYLIP files are always output in non-interleaved format but taxon names can be printed in relaxed or strict format (i.e., with unlimited or limited signs). The default information file "FcC_info.xls" also reports base compositions for nucleotide data of single and supermatrix files while an additional info file "FcC_structure.txt" lists single loop as well as stem pairing positions if structure sequences in dot-bracket format are present. Concatenated and/or single infile structure sequences are printed to this file too. For more details about FASconCAT-G usage and options, see the 'Usage/Options' section.

2 Usage/Options

In order to run FASconCAT-G, open the terminal of your operating system and change directories to the folder where FASconCAT-G and your input files are located. Type the name of your FASconCAT-G version, followed either by a blank and your demand options in one row to start FASconCAT-G directly or just hit <enter> to open the FASconCAT-G menu. Notice that all input files have to be located in the FASconCAT-G home folder. To execute FASconCAT-G, a Perl interpreter must be installed on the current run system. Linux and Mac systems typically come with an interpreter pre-installed while Windows users will have to install a Perl interpreter (e.g., post). We recommend the ActivePerl interpreter, which can be downloaded for free here:

- http://activeperl.softonic.de/

2.1 Start FASconCAT-G via menu

2.1.1 Open the menu under Windows

Open a prompt (DOS) terminal on your Windows system and navigate to the folder where FASconCAT-G and files are located <cd your_path. . . >. Then open FASconCAT-G:

- C:\FASconCAT-G_Folder> FASconCAT-G_v1.0.pl <enter>

2.1.2 Open the menu under Linux/Mac

Open a terminal and navigate to the folder where FASconCAT-G and files are located <cd your_path. . . >. Then open FASconCAT:

- `user@user:\~`/FASconCAT-G_Folder> perl FASconCAT-G_v1.0.pl <enter>

Note: in some cases Linux/Mac users got a path error report from Perl. In this case, the executable for Perl and all of its associated files are located in another directory as /usr/bin/ (e.g. /usr/local/bin/). To start FASconCAT-G without adding the full directory path to the perl start command (e.g. /usr/local/bin/perl FASconCAT-G_v1.0.pl) users can change the default path directory to the perl executable in the first line of the FASconCAT-G script by just opening FASconCAT-G with an text editor and changing the first line of the script:

- line 1: #!usr/bin/perl ; ↪ #!usr/local/bin/perl ;

2.1.3 Menu handling

The main menu of FASconCAT-G is subdivided into two parts separated by a dashed line. The upper portion displays all possible options and their associated commands for adjustment. The lower part shows the current parameter settings of FASconCAT.

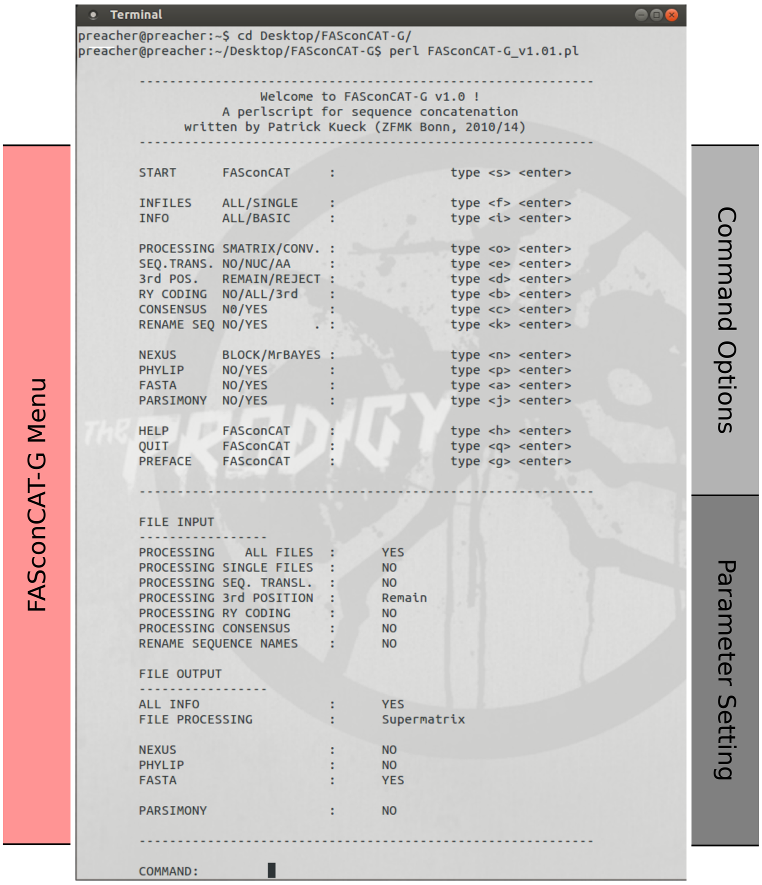

Figure 1: Menu of FASconCAT

To change the default parameter setting type the option associated command into the command line and press <enter>. The new setting configuration will be displayed in the lower part of the menu. After finishing parameter configuration FASconCAT-G can be started by typing “s” and pressing <enter>. For getting help type “h” and press <enter>, to return to FASconCAT-G press <enter>, to quit the program type “q” and press <enter>.

2.2 Start FASconCAT-G via single command line

FASconCAT-G can be directly started by single line commands, which simplifies the implementation of FASconCAT-G into complex analyses pipelines. Change directories to the folder where FASconCAT-G and your input files are located and then type the name of the FASconCAT-G version followed by a blank and the desired command options with a minus (-) sign in front of each. Then press <enter>. Make sure you write the input options correctly, for example “-i” and not “- i”. Otherwise FASconCAT-G will not start working but instead open the menu.

- C:\FASconCAT-G_Folder> perl FASconCAT-G_v1.0.pl -help <enter> $\hookrightarrow$ help menu
- C:\FASconCAT-G_Folder> perl FASconCAT-G_v1.0.pl -s <enter> $\hookrightarrow$ start FASconCAT-G under default options.

Table 1: Overview of option codes via single command line start

**Info options**	**Command**			
Help menu	-help			
Preface	-g			
Start	-s			
**Parameter options**				**Default**
Defined Input files	-f			All Input Files
Print Limited Info	-i			Print All Info
File Conversion	-o			Supermatrix Output
File Conversion & Supermatrix Output	-o	-o		”
Translation Nucleotide to Amino Acid	-e			none
Translation Amino Acid to Nucleotide	-e	-e		”
Reject 3rd Codon Position	-d			none
RY Coding Complete Sequence	-b			none
RY Coding 3rd Codon Position	-b	-b		”
Create ’Frequency’ Consensus Sequence(s)	-c			none
Create ’Majority’ Consensus Sequence(s)	-c	-c		”
Create ’Strict’ Consensus Sequence(s)	-c	-c	-c	”
Print Parsimony Informative Sites	-j			none
Rename Sequence Names	-k			none
Reject FASTA Output	-a			Print FASTA
Print PHYLIP Output (Strict)	-p			none
Print PHYLIP Output (Relaxed)	-p	-p		”
Print NEXUS Output (Block)	-n			none
Print NEXUS Output (MrBayes)	-n	-n		”
Print Partition File	-l			”
Start ProtTest for RAxML Partition File	-m			”

2.3 Options

FASconCAT-G has many options that can either be invoked in the program menu or via a single command line. Note: When invoking options in the single command line, use a minus sign before the command (e.g., "-i"). While in the working menu, simply type the command (e.g., "i") and hit <enter>.

2.3.1 File Processing (-o option)

The file processing option (-o option) is the main option in FASconCAT-G. With this option the user specifies if single alignment infiles are either converted, concatenated, or converted & concatenated by FASconCAT-G. Note: single alignment infiles do not need to be in the same file format as FASconCAT-G can handle any combination of acceptable infile formats. Under default file processing setting (Supermatrix), FASconCAT-G generates a concatenated supermatrix from given infiles in selected output format(s) (e.g., FcC_supermatrix.fas). If only one infile is defined under the 'Supermatrix' option, FASconCAT-G converts the selected infile to the selected output format.

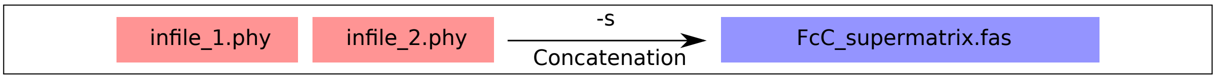

Figure 2: Example usage 1 of file processing. Concatenation of PHYLIP formated infiles to a FASTA formated supermatrix outfile (using default options).

To convert multiple infiles to different output formats without sequence concatenation, use the "-o'" command to change the file processing option to "Convert" (File Processing: Convert).

- perl FASconCAT-G_v1.0.pl -o -s <enter> ↪ file Conversion under default options.

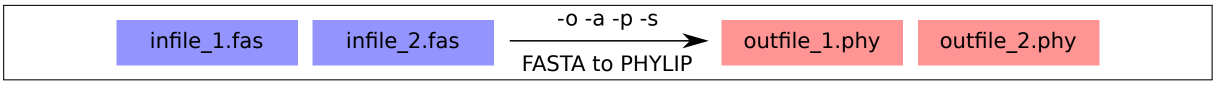

Figure 3: Example usage 2 of file processing. Conversion of FASTA formated infiles to PHYLIP (strict) formated outfiles.

To convert and simultaneously concatenate multiple infiles to different output formats, use the "-o -o" command (File Processing: Supermatrix/Convert).

- perl FASconCAT-G_v1.0.pl -o -o -s <enter> ↪ file Conversion & sequence concatenation under default options.

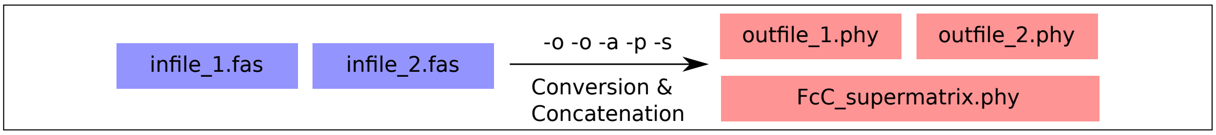

Figure 4: Example usage 3 of file processing. Conversion & Concatenation of FASTA formated infiles to PHYLIP (strict) formated outfiles.

2.3.2 Sequence Translation (-e option)

FASconCAT-G can translate standard nucleotide sequence states to amino acid characters and visce versa. If sequence translation is defined, the translation process will be the first of all defined process steps conducted by FASconCAT-G. FASconCAT-G can recognize and handle amino acid and nucleotide data sets in a single process run. Therefore, FASconCAT-G only translates sequences of infiles which are suitable for a defined translation process. This makes it very easy to concatenate a mixture of different infile sequence types to one specific supermatrix sequence type (Figure 5). Under default, the translation option is set to 'none'.

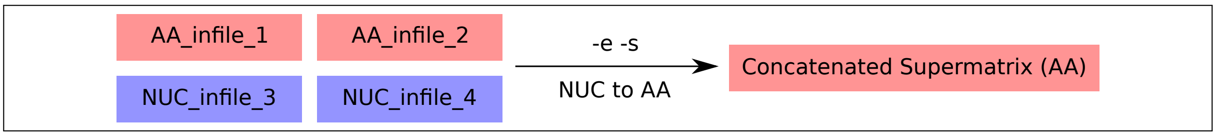

Figure 5: Example usage 1 of sequence translation. Concatenation of a mixture of different infile sequence types to one specific supermatrix sequence type (NUC: nucleotide data, AA: amino acid data).

To translate nuceotide triplet states to amino acid characters use the “-e” command. FASconCAT-G does not recognize start or stop codons of nucleotide data. It will recode triplets based on the first sequence position until the last sequence position is reached. For sequence translation of nucleotide data FASconCAT-G uses the standard IUPAC triplet codes for amino acid characters. FASconCAT-G will print an info text on the terminal if sequence lengths are not a multiple of three, but will not abort the translation process. Instead, FASconCAT-G will translate incomplete nucleotide triplets to ’?’. FASconCAT-G translates nucleotide triplets even if triplets contain ambiguity codes, provided that the triplets are still assignable to specific amino acid characters (e.g. ’YTR’ ↪ Leucine/L). Otherwise, unspecific triplets are translated to ’?’ (e.g. ’RCT’ ↪ ?).

- perl FASconCAT-G_v1.0.pl -e -s <enter> ↪ translating nucleotide character states to amino acid character states under default options.

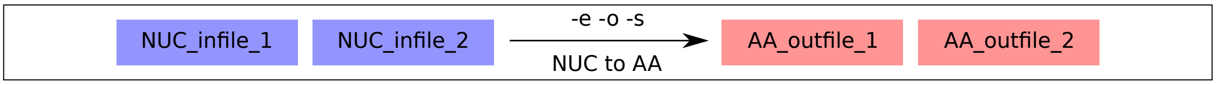

Figure 6: Example usage 2 of sequence translation. Conversion of nucleotide infiles to amino acid outfiles (NUC: nucleotide data, AA: amino acid data).

To translate amino acid characters to nucleotide triplet states use the “-e -e” command. Amino acid characters are translated to nucleotide triplets using the compressed IUPAC triplet code for a given amino acid character (e.g. Phenylalanin/F ↪ ’TTY’). Unrecognized sequence characters in amino acid sequences like ’-’ or ’X’ are translated to ’???’. Note: stop codon signs are not allowed in FASconCAT-G and have to be excluded before file processing! FASconCAT-G does not check for the correctness of given reading frames!

- perl FASconCAT-G_v1.0.pl -e -e -s <enter> ↪ translating amino acid character states to nucleotide character states under default options.

2.3.3 Renaming Sequence Names (-k option)

FASconCAT-G can rename defined sequence names prior to file processing. The user must provide an extra info file named “new_seq_names.txt” in the FASconCAT-G home folder, which in each row lists the old name delimited from the new name by a tabstop (Table 2). Otherwise, FASconCAT-G will abort with an error prompt. Sequences which are not listed in “new_seq_names.txt” are left unchanged. FASconCAT-G will print additional information of the sequence renaming process to a new outfile named “FcC_rename_control.txt”. Note: the new defined sequence names in “new_seq_names.txt” must be unique. Otherwise, FASconCAT-G will abort with an error prompt.

Table 2: Example format of user defined infile (“new_seq_names.txt”) for sequence renaming.

**Given Sequence Names**		**New Defined Sequence Names**
Given_Name_sequence_1	<tabstop>	New_Name_sequence_1
Given_Name_sequence_2	<tabstop>	New_Name_sequence_2
Given_Name_sequence_3	<tabstop>	New_Name_sequence_3
Given_Name_sequence_n	<tabstop>	New_Name_sequence_n

2.3.4 Rejection of 3rd Nucleotide Codon Positions (-d option)

To reject each third codon position in nucleotide data use the “-d” command. FASconCAT-G will reject each third sequence position of given nucleotide infiles and proceed with reduced sequences with respect to other defined FASconCAT-G options. Amino acid sequences will not be reduced under the “-d” command. To reduce amino acid sequences as well, use the sequence translation option (“-e” command) together with the “-d” command. FASconCAT-G will translate amino acid states to corresponding nucleotide characters using compressed IUPAC codes and afterwards exclude each third position in translated sequences (Figure 7).

- perl FASconCAT-G_v1.0.pl -d -s <enter> $\hookrightarrow$ rejecting each 3rd nucleotide state under default options.

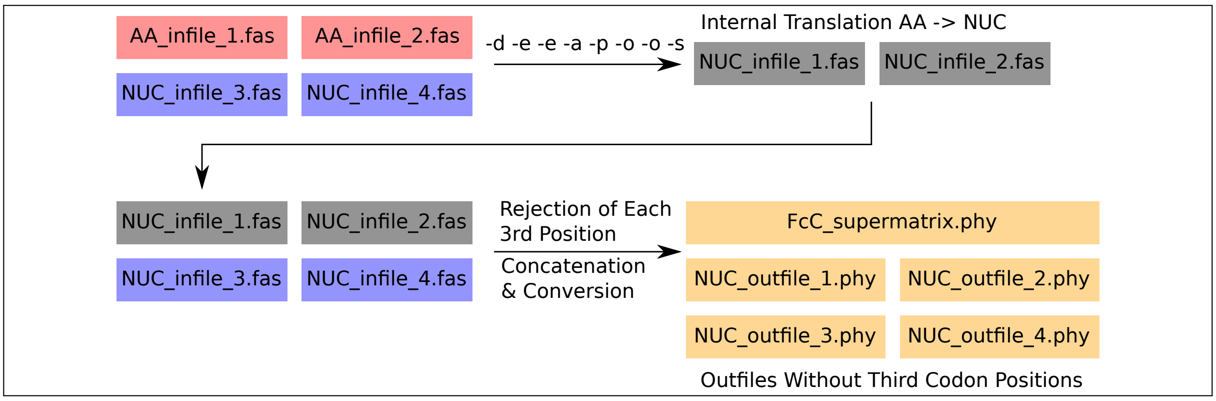

Figure 7: Example usage 1 of 3rd nucleotide codon position exclusions. First, amino acid sequences of given FASTA infiles are internally translated to nucleotide triplets using the “-e -e” command. Afterwards, each third sequence position of given infiles is rejected by FASconCAT-G (“-d” command). Finally, reduced sequences of given infiles are converted and printed as PHYLIP (strict) formated outfiles (“-a -p” command) and, due to the “-o -o” command, concatenated and printed as PHYLIP (strict) formated supermatrix outfile as well.

Note: concatenation of different infile sequence types (e.g., nucleotide and amino acid sequences) without the “-e” command will lead to a supermatrix with different ranges of nucleotide and amino acid character states (Figure 8).

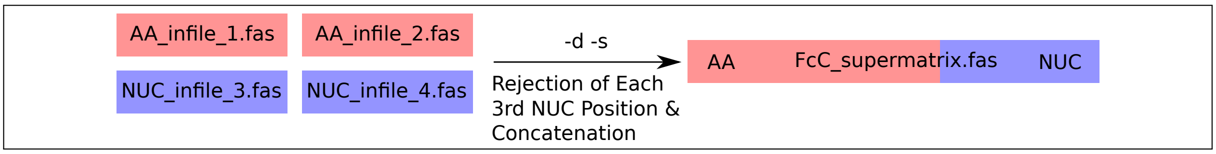

Figure 8: Example usage 2 of 3rd nucleotide codon position exclusions. Third sequence positions are only excluded in nucleotide infiles. Therefore, the concatenated supermatrix consists of reduced nucleotide sequences and unreduced amino acid sequences.

2.3.5 RY Coding (-b option)

RY coding can either be applied to each third nucleotide sequence position (“-b” command) or to complete nucleotide sequences (“-b -b” command). The R code is used for purine states (A or G $\hookrightarrow$ R) while the Y code is used for pyrimidines (C or T|U $\hookrightarrow$ Y). Amino acid sequences are left unchanged, except when the “-e -e” option has been chosen (see section 2.3.3 and Figure 7).

- perl FASconCAT-G_v1.0.pl -b -s <enter> $\hookrightarrow$ RY coding of 3rd sequence positions in nucleotide sequences under default options.
- perl FASconCAT-G_v1.0.pl -b -b -s <enter> $\hookrightarrow$ RY coding of complete nucleotide sequences under default options.

2.3.6 Create Consensus Sequences (-c option)

FASconCAT-G can create consensus sequences for matching defined sequence blocks within given infiles (see Figure 9, 10). Sequence blocks are matched by identical alphanumeric characters before the first underscore (case sensitive) in sequence names. If no underscore is present, the complete sequence name will be used for sequence block identification. Consensus sequences of defined sequence blocks are named by the common sequence name prefix and an additional name suffix ('_consensus') (see Table 3). In sequence concatenation processes, missing sequence blocks in given infiles are replaced by the corresponding replacement variables for the sequence type ('N' for nucleotide data, 'X' for amino acid data) in the supermatrix out file.

Table 3: Example of defined consensus sequence blocks. Sequence blocks are identified by identical alphanumeric characters of sequence names before the first underscore. If an underscore is not given, complete sequence names will be used for consensus block identification. Consensus sequences of defined sequence blocks are named by the corresponding block specified alphanumeric characters and the suffix '_consensus'.

Sequences of a Given Infile		Consensus Sequence Blocks	
Taxon_1	AGGGCCCTTG...		
Taxon_2_allel_2	AGGGCCCTTG...		
Taxon	AGGGCCCTTG...	Taxon_consensus	AGGGCCCTTG...
Taxon1_allel_1	AGGGCCCTTG...	Taxon1_consensus	AGGGCCCTTG...
Tax_1	AGGGTTTTTG...		
Tax_2	AGGGTTTTTG...	Tax_consensus	AGGGTTTTTG...

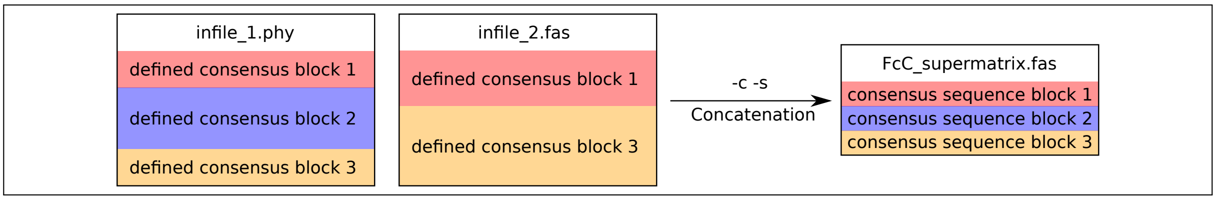

Figure 9: Example usage 1 of the consensus sequence option for infile concatenation. Two infiles each with defined sequence blocks are concatenated into a supermatrix outfile. The supermatrix outfile contains a single consensus sequence for matching defined sequence blocks within each infile. If infile_1.phy and infile_2.fas share defined sequence blocks, the consensus sequence from each infile will be concatenated in the FcC_supermatrix.fas file. Missing sequence blocks are replaced by corresponding replacement variables ('N' for nucleotide data, 'X' for amino acid data) in the concatenated supermatrix file.

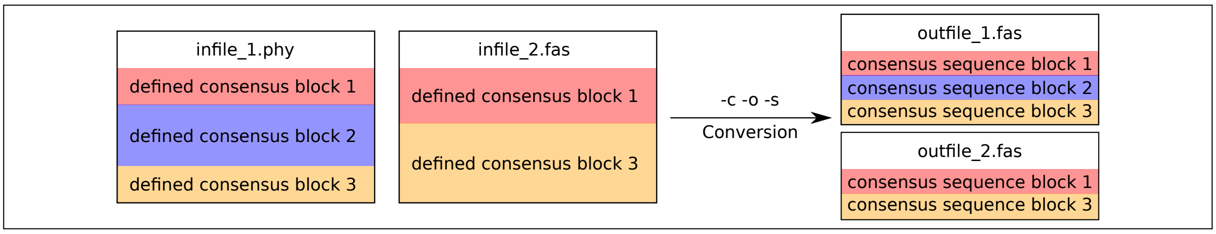

Figure 10: Example usage 2 of the consensus sequence option for infile format conversion. In this case, infille_1.phy and infile_2.fas are converted to FASTA files. Matching sequence blocks within each infile are output as a consequence sequence.

Three different consensus types are available in FASconCAT-G to process consensus sequences of defined sequence blocks: 'Most Frequent Consensus' ("-c" command), 'Majority Rule Consensus' ("-c -c" command), and 'Strict Consensus' ("-c -c -c" command).

'Most Frequent Consensus'. The 'Most Frequent Consensus' option considers the most frequent character state among defined sequence blocks as the consensus character state. If two or more character states are equally frequent, FASconCAT-G uses either the corresponding IUPAC ambiguity code as the consensus character state (nucleotide data) or '?' (amino acid data and nucleotide data) (Table 4).

- perl FASconCAT-G_v1.0.pl -c -s <enter> ↪ create 'Most Frequent Consensus' sequences of defined sequence blocks under default options.

Table 4: Example of a most frequent consensus block. Most frequent character states of a specific site position are used as consensus character state. If two or more character states are equally frequently distributed, FASconCAT-G takes the corresponding IUPAC ambiguity code (if appropriate) or a '?' as consensus character state.

**Sequences of a Given Nucleotide Infile**							**Sequences of a Given Amino Acid Infile**						
tax1_allel1	A	G	-	G	C	C	tax1_allel1	A	L	L	R	?	-
tax1_allel2	A	G	-	G	C	C	tax1_allel2	A	L	L	R	X	-
tax1_allel3	A	C	?	C	-	-	tax1_allel3	A	-	L	-	X	-
tax1_allel4	T	N	-	C	T	-	tax1_allel4	A	R	R	Y	X	H
tax1_allel5	T	N	-	C	T	-	tax1_allel5	A	R	R	X	Y	H
tax1_consensus	A	N	-	C	Y	-	tax1_consensus	A	?	L	R	X	-

'Majority Rule Consensus'. The 'Majority Rule Consensus' option considers character states which occur at a given site position in more than 50% of sequences of a defined sequence block as consensus character state. Otherwise, FASconCAT-G uses a '?' as consensus character state (Table 5).

- perl FASconCAT-G_v1.0.pl -c -c -s <enter> ↪ create 'Majority Rule Consensus' sequences of defined sequence blocks under default options.

Table 5: Example of a majority rule consensus block. Character states of a specific site position which occure in more than 50% of sequences in a defined sequence block are used as consensus character state. Otherwise, FASconCAT-G takes a '?' as consensus character state.

**Sequences of a Given Nucleotide Infile**							**Sequences of a Given Amino Acid Infile**						
tax1_allel1	A	G	-	G	C	C	tax1_allel1	A	L	L	R	?	-
tax1_allel2	A	G	-	G	C	C	tax1_allel2	A	L	L	R	X	-
tax1_allel3	A	C	?	C	-	-	tax1_allel3	A	-	L	-	X	-
tax1_allel4	T	N	-	C	T	-	tax1_allel4	A	R	R	Y	X	H
tax1_allel5	T	N	-	C	T	-	tax1_allel5	A	R	R	X	Y	H
tax1_consensus	A	?	-	C	?	-	tax1_consensus	A	?	L	?	X	-

'Strict Consensus'. The 'Strict Consensus' option considers all character states at a given site position to generate a strict consensus sequence for a defined sequence block using IUPAC ambiguity codes for nucleotide data and a 'X' for amino acid data (for nucleotide data, '-' and '?' are ignored as long as a nucleotide character state exists for a specific site position). However, if a specific site position of a defined sequence block lacks a specific character state and consists of only gap states ('-') and missing data states ('?'), FASconCAT-G will output a '?' as the consensus character state (Table 6).

- perl FASconCAT-G_v1.0.pl -c -c -c -s <enter> ↪ create 'Strict Consensus' sequences of defined sequence blocks under default options.

Table 6: Example of a strict consensus block. A consensus character will only be assigned when the character state at given site is unanimous in all sequences of a defined sequence block. In all other cases, FASconCAT-G will assign the appropriate IUPAC ambiguity code for nucleotide states ('-' and '?' are not considered) or 'X' for amino acid data. If a site position consists of only gaps ('-') and missing data ('?'), FASconCAT-G will assign '?' as the consensus character state.

**Sequences of a Given Nucleotide Infile**							**Sequences of a Given Amino Acid Infile**						
tax1_allel1	A	G	-	G	C	C	tax1_allel1	A	L	L	R	?	-
tax1_allel2	A	G	-	G	C	C	tax1_allel2	A	L	L	R	X	-
tax1_allel3	A	C	?	C	-	-	tax1_allel3	A	-	L	-	X	-
tax1_allel4	T	N	-	C	T	-	tax1_allel4	A	R	R	Y	X	H
tax1_allel5	T	N	-	C	T	-	tax1_allel5	A	R	R	X	Y	H
tax1_consensus	W	N	?	S	Y	C	tax1_consensus	A	X	X	X	X	X

2.3.7 Handling of Defined Input Files (-f option)

When the defined input files option "-f " is invoked, FASconCAT-G requires the user to define infiles for processing. The program will list files in FASTA (.fas), PHYLIP (.phy) and CLUSTAL (.aln) format currently found in its home folder along with an associated list number (Table 7). So the user can define specific files for defined processing steps. Type the file associated number of selected files separated by comma without blanks in one row and press <enter>. By typing b and <enter>, FASconCAT-G will skip back to the main menu. Note: the "-f" command is unsuitable for pipeline processes, because it requires a user sepcified input list of infiles for FASconCAT-G.

Table 7: Example list of available infiles for FcC processing when option "-f " is invoked.

**Listnumber**	**Filename**
0	example_file_1.fas
1	example_file_2.phy
2	example_file_3.aln
3	example_file_4.aln
4	example_file_5.fas

- 1st COMMAND: -f -s <enter> ↪ start FASconCAT-G under default and displays available defined infiles
- 2nd COMMAND: 2,3,4 <enter> ↪ only the defined infiles associated with list number 2,3, and 4 of table 7 will be processed

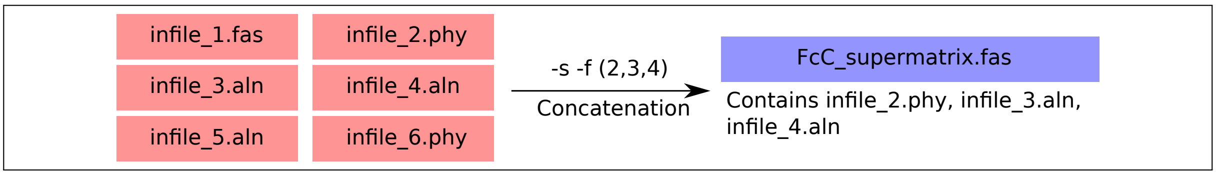

Figure 11: Example usage of the defined infile option ”-f”. Using the “-f” command, FASconCAT-G will print a list of potentially infiles identified from the FASconCAT-G home folder. In this example infiles 2,3, and 4 have been selected from the FASconCAT-G infile list for the default sequence concatenation process.

2.3.8 Print OUT of Parsimony Informative Sites (-j option)

FASconCAT-G will print OUT additional information file(s) identifying parsimony-informative sites of given infiles and/or the concatenated supermatrix (depending on the defined “-o” command) if the user invokes the ”-j” command. A site is parsimony-informative if it contains at least two types of nucleotides (or amino acids), and at least two of them occur with a minimum frequency of two. The file format of parsimony-informative alignment files depends on the chosen output format(s) (Figure 13).

- COMMAND: -j -s <enter> ↪ start FASconCAT-G under default with additional supermatrix print OUT of parsimony informative sites

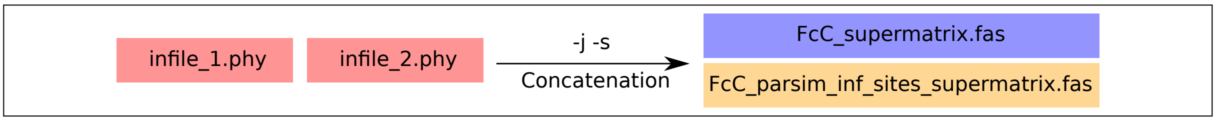

Figure 12: Example usage 1 of parsimony informative site print OUT command “-j”. In this example, FASconCAT-G will print an additional outfile that contains parsimony-informative sites of the concatenated supermatrix outfile.

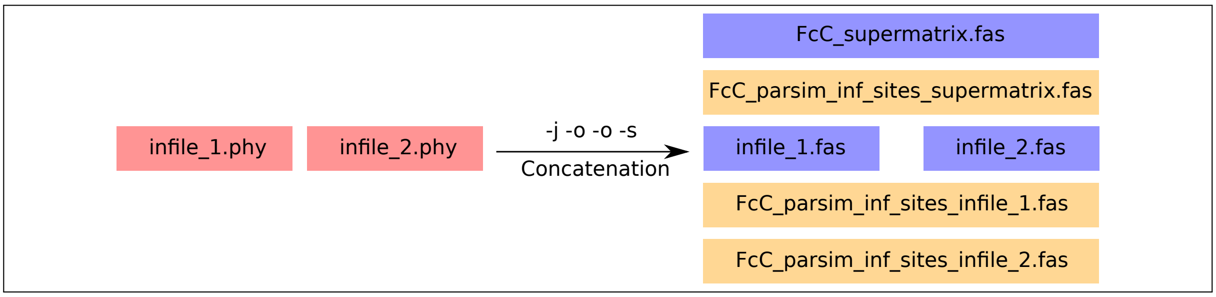

Figure 13: Example usage 2 of parsimony informative site print OUT command “-j”. In this example, FASconCAT-G will print additional outfiles that contain parsimony-informative sites of both the concatenated supermatrix outfile as well as the FASTA converted infiles (combined with “-o -o” option).

2.3.9 Print OUT Partition File(s) (-l option)

With the “-l” command invoked FASconCAT-G generates additional gene partition output files if the sequence concatenation option has been defined. If the user selects to output a supermatrix in FASTA and/or PHYLIP format, FASconCAT-G prints an associated gene partition file (“FcC_supermatrix_partition.txt”) which can be directly used for a Maximum Likelihood analysis with RAxML. Without using the ProtTest option (“-m”) FASconCAT-G chooses the LG substitution model as default model for given or translated amino acid gene partitions. For a supermatrix output in NEXUS (block) format, FASconCAT-G prints an additional NEXUS file in which single gene partitions are defined as single sequence blocks (“FcC_supermatrix_partition.nex”). If the NEXUS (MrBayes) format is defined, FASconCAT-G prints an additional MrBayes executable file with defined partitions (also “FcC_supermatrix_partition.nex”). The

parameters in the MrBayes partitioned executable NEXUS file vary from the non-partitioned version (Table 8). Note: The LG model fits in most cases, but must NOT be mandatory the best fitting model for given data! To identify the best fitting amino acid substitution model for each gene partition we suggest to use FASconCAT-G together with the external ProtTest software (see “-m option”).

Table 8: Overview of the MrBayes parameters integrated into the NEXUS output under the “-n -n” option in combination with the “-l” option.

MrBayes Commands	Set Up
Number of generations	10000000
Print frequency	1000
Sample frequency	1000
Number of chains	4
Diagnostic frequency	1000
Diagnostic statistic	maxstddev
Save branch lengths	yes
Checkpointing	100000
unlink shape	all
pinvar	all
statefreq	all
revmat	all
prset applyto	all
ratepr	variabel
lset applyto	all
temp	0.1
Number of substitution	6
Rates	invgamma

2.3.10 Using ProtTest for Amino Acid Model Specification (-m option)

FASconCAT-G offers the option to generate the best-fit protein model for each amino acid gene partition in RAxML partition formatted supermatrices using the external software, ProtTest version 3.3 (Darriba et al. (2011), ProtTest 3: fast selection of best-fit models of protein evolution, Bioinformatics). ProtTest will only be executed for given or translated amino acid infiles if sequence concatenation has been chosen together with the partition print option (“-l”), but not for supermatrices in NEXUS format. FASconCAT-G uses default settings for ProtTest version 3.3 and the best-fit model will be selected by the ProtTest BIC criterium. Note: all outfiles from ProtTest version 3.3 are placed in the FAScoCAT-G home directory. FASconCAT-G will print a warning message to the output terminal if ProtTest could not be executed successfully. In that case, FASconCAT-G will assign the LG model for the corresponding gene partition in the RAxML gene partition file. ProtTest version 3.3 can be downloaded for free here:

- `http://www.mybiosoftware.com/phylogenetic-analysis/2854`

The name of the implemented ProtTest version is ”prottest-3.3.jar”. FASconCAT-G can only start ProtTest executables with that name. To change the default ProtTest executable name, just open FASconCAT-G with an text editor and change the ProtTest name in line 10. Be aware that the ProtTest name is surrounded by single quotation marks:

- line 10: my $prottest = ’prottest-3.3.jar’ ; ↪ my $prottest = ’NEW_PROTTEST_NAME’ ;

2.3.11 Switch Off Print OUT File(s) in FASTA format (-a option)

Under default, FASconCAT-G prints OUT all defined outfile(s) in FASTA format (.fas). Use the “-a” command to switch off the FASTA format print OUT. Note: if the FASTA format print OUT has been switched off and no other output format has been defined, FASconCAT-G will abort with an error prompt. Therefore, we strongly recommend to use the “-a” command via the terminal command line only in combination with another file format print OUT command (e.g. “-p” or “-n”) (Figure 14). This note is especially important if FASconCAT-G should be implemented in automated pipeline processes.

- COMMAND: -a -s <enter> ↪ abort FASconCAT-G due to non-specified output formats.
- COMMAND: -a -p -s <enter> ↪ start FASconCAT-G under default wih PHYLIP output format instead of FASTA output format.

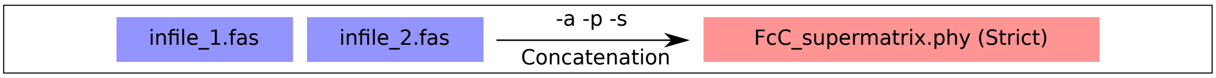

Figure 14: Example usage of non-FASTA formated print OUT command “-a”. In this case, FASconCAT-G will print OUT the concatenated supermatrix in PHYLIP (strict) format. Be aware, FASconCAT-G will abort with an error prompt if no other outfile format has been chosen.

2.3.12 Print OUT File(s) in NEXUS format (-n option)

FASconCAT-G will output defined outfiles in NEXUS (block) format with the “-n” command invoked (Figure 15), which can be directly loaded into programs such as PAUP or MrBayes. Alternatively, FASconCAT-G can generate an executable NEXUS file for the bayesian analysis software MrBayes with the “-n -n” command (Figure 16). The MrBayes executable is printed with a set of parameters that serve as good starting point for bayesian analyses but these can easily be changed with any text editor. If a structure string in dot-bracket format is given while dots code unpaired (loop) positions and brackets code pairings (stems) (“(” ↪ opening a stem, “)” ↪ closing a stem), FASconCAT-G compiles a partition set for MrBayes with single charset for stem and loop regions. Table 9 gives an overview of the integrated set up for MrBayes.

- COMMAND: -n -s <enter> ↪ start FASconCAT-G under default with additional supermatrix print OUT in NEXUS (block) format
- COMMAND: -n -n -s <enter> ↪ start FASconCAT-G under default with additional supermatrix print OUT in NEXUS (MrBAYES) format

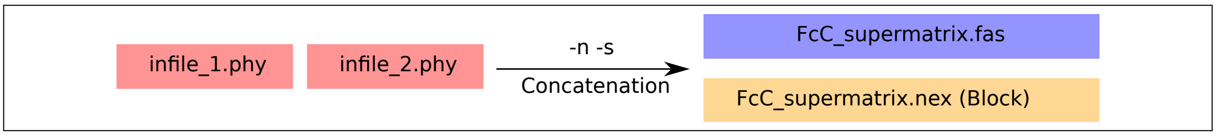

Figure 15: Example usage 1 of NEXUS (block) formatted print OUT command “-n”. In this example, FASconCAT-G will print a NEXUS (block) formatted version of the concatenated supermatrix outfile in addition to the FASTA supermatrix outfile (the default FASTA format print OUT has not been switched off).

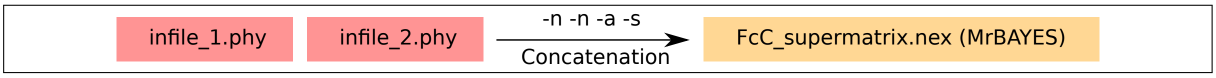

Figure 16: Example usage 1 of NEXUS (MrBayes) formated print OUT command “-n -n”. In this example, FASconCAT-G will only print a NEXUS (MrBayes) formatted version of the concatenated supermatrix (the default FASTA format print OUT has been switched off by the “-a” command).

Table 9: Overview of the MrBayes parameters integrated into the NEXUS output under the “-n -n” option. Structure partition parameters are only printed out by given structure information.

**MrBayes Commands**	**Set Up**
Number of generations	2000
Print frequency	100
Sample frequency	100
Number of chains	4
Save branch lengths	yes
Set autoclose	yes
No warnings	yes
Unlink statefrequency	all
tratio	all
Shape	all
Number of substitution	6
Rates	gamma
Sump burnin	20
Number of sump runs	2
Sumt burnin	20
Number of sumt runs	2
Inputfilename	FcC_smatrix.nex
**Structure Partition**	
Set partition	looms
partition looms	2: loops, stems
lset 1	nucmodel= 4by4
lset 2	nucmodel= dublet

2.3.13 Print OUT File(s) in PHYLIP format (-p option)

With the “-p” or the “-p -p” command invoked FASconCAT-G generates interleaved PHYLIP (.phy) output files in strict format (taxon name restriction up to 10 signs) or relaxed format (no restriction in character number for taxon names), respectively. To switch off the FASTA formatted print OUT (default setting for print OUT), enter the “-a” command as well (Figure 17) (see section 2.3.10).

- COMMAND: -p -s <enter> ↪ start FASconCAT-G under default with additional supermatrix print OUT in PHYLIP (strict) format
- COMMAND: -p -p -s <enter> ↪ start FASconCAT-G under default with additional supermatrix print OUT in PHYLIP (relaxed) format
- COMMAND: -p -a -s <enter> ↪ start FASconCAT-G with supermatrix print OUT only in PHYLIP (strict) format

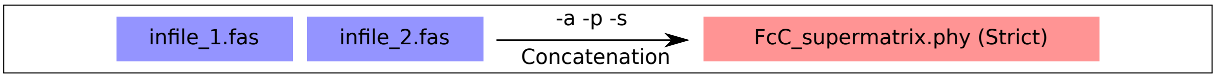

Figure 17: Example usage 1 of PHYLIP (strict) formatted print OUT “-p” command. In this case, FASconCAT-G will only print OUT a concatenated supermatrix outfile in PHYLIP (strict) format because the “-a” command (switches off the default FASTA formatted print OUT of defined outfile) is also invoked.

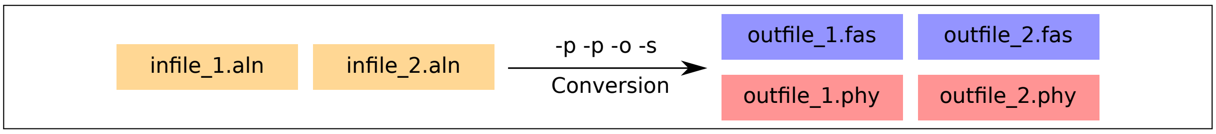

Figure 18: Example usage 2 of PHYLIP (relaxed) formatted print OUT “-p -p” command. In this example, FASconCAT-G will convert the CLUSTAL infiles and print OUT outfiles in both PHYLIP (relaxed) and FASTA format.

2.3.14 Reduction of Information Print OUT (-i option)

By default FASconCAT-G provides useful information about the supermatrix output file and all single input files (Table 10). However, the evaluation of this additional information often results in longer computation times depending on the data set. The user can therefore invoke the “-i” command to increase the overall computation speed by decreasing information sampling and print out a reduced information file (Table 11).

- COMMAND: -i -s <enter> ↪ start FASconCAT-G under default with reduced information print OUT

Table 10: List of default information content of the FASconCAT-G FcC_info.xls outputfile

Default Information	Supermatrix File	Converted Infiles
Defined Parameter Settings	yes	yes
Single Fragment Ranges	yes	no
Sequence Type	yes	yes
Alignment Length	yes	yes
Total Number of Sequences	yes	yes
Total Number of Character States	yes	yes
Total Number of Nucleotide States	yes	yes
Total Number of Amino Acid States	yes	yes
Total Number of Gap States	yes	yes
Total Number of Ambiguity States	yes	yes
Total Number of Missing States	yes	yes
Total Number of GC Nucleotides	yes	yes
Total Number of Parsimony Informative Sites	yes	yes
Total Number of Missing Sequences per Fragment	yes	no
Total Number of Given Sequences per Fragment	yes	no
Total Number of Concatenated Sequences per Fragment	yes	no
Percentage of Nucleotide Character States	yes	no
Percentage of Amino Acid Character States	yes	no
Percentage of Gap States	yes	no
Percentage of Ambiguity States	yes	no
Percentage of Missing States	yes	no
Percent GC Content	yes	no
Percentage of Parsimony Informative Sites	yes	no
Percent & Total Number of Loop Character States	yes	no
Percent & Total Number of Stem Character States	yes	no

Table 11: List of reduced information content of the FASconCAT-G FcC_info.xls outputfile

**Reduced Information ("-i" Command)**	**Supermatrix File**	**Converted Infiles**
Defined Parameter Settings	yes	yes
Single Fragment Ranges	yes	no
Alignment Length	yes	yes
Total Number of Missing Sequences per Fragment	yes	no
Total Number of Given Sequences per Fragment	yes	no
Total Number of Concatenated Sequences per Fragment	yes	no

3 Internals

3.1 Input/Output

FASconCAT-G is able to import three different file formats. The format of the output files depend on the user determined parameter settings. Table 12 shows possible input and output formats while Table 13, 14, 15 show examples of correct infile formats. Note: The outgroup specification in the MrBayes NEXUS partition file is commented out by square brackets ("[outgroup outgroupname;]") To define an outgroup sequence remove the square brackets in that line and replace "outgroupname" with desired outgroup sequence name.

Table 12: Overview of possible input and output formats under given parameter options.

**Input Format**	**Ending**	
FASTA	.fas/.fasta	
PHYLIP	.phy	
CLUSTAL	.aln	
**Output Format**	**Ending**	**Output Options**
FASTA	.fas	default (-a to switch off)
PHYLIP (Strict)	.phy	-p
PHYLIP (Relaxed)	.phy	-p -p
NEXUS (Block)	.nex	-n
NEXUS (MrBayes)	.nex	-n -n
**Output files**	**Content**	
FcC_supermatrix.xxx	Supermatrix in FASTA format	
FcC_*infile*.xxx	Converted outfile of given *infile*	
FcC_info.xls	Sequence info	
FcC_parsim_inf_sites_supermatrix.xxx	Parsimony informative supermatrix sites	
FcC_parsim_inf_sites_*infile*.xxx	Parsimony informative sites of given *infile* after file conversion	
FcC_structure.txt	Secondary structure info	
FcC_rename_control.txt	Info about the sequence renaming process	

Table 13: Examples of FASTA formats in non-interleaved (Format 1) and interleaved format (Format 2).

**FASTA Format 1**
>Name_sequence_1
AGCTCCCGTCCTTTG–AGA–GTGTCCTTTCCT
>Name_sequence_2
AGCTCCGGCCCTTTG–AGA–GTGTCCTTTCCT
>Name_sequence_n
AGCTCCCGTCCTTTGGAGAGGTGTCCTTTCCT

**FASTA Format 2**
>Name_sequence_1
AGCTGTCCTTTCTTG–AGA–GTGTCCTTTCCT
GGGGCCCTTTC–GGTTTTCCCCGTCCTTTCCT
>Name_sequence_n
AGCTGTCCTTTCTTGCAGACGTGTCCTTTCCT
GGGGCTTCAAGTTTTCCCCCGGGTCCTTTCCT

Table 14: Example of a CLUSTAL formatted input file.

**CLUSTAL format**	
CLUSTAL X (1.81) multiple sequence alignment	
<line break>	
<line break>	
Name_sequence_1	AGGGCCCTTGCGCTTGCTTCC
Name_sequence_2	AGGGCCCTTGCGCCTGCTTCC
Name_sequence_n	AGGCCCCTTGCGCCGGCTTCC
<line break>	
<line break>	
Name_sequence_1	ATTTCCCTTGGGCTTGCTTCC
Name_sequence_2	ATTTCCCTTGGGCCTGCTTCC
Name_sequence_n	ATCTCCCTTGGGCCGGCTTCC

Table 15: Examples of PHYLIP infile formats in non-interleaved (Format 1) and interleaved format (Format 2).

**PHYLIP Format 1**		
6 40		
Name_sequence_1	AGGGCCCTTGCGCTTGGCCC	
Name_sequence_2	AGGGCCCTTGCGCCTCCCCC	
Name_sequence_n	AGGCCCCTTGCGCCGCCCGG	
**PHYLIP Format 2**		
6 40		
Name_sequence_1	AGGGCCCTTG	CGCTTGGCCC
Name_sequence_2	AGGGCCCTTG	CGCCTCCCCC
Name_sequence_n	AGGCCCCTTG	CGCCGCCCGG
<line break>		
	ATTTCCCTTG	GGCTTCCCCC
	ATTTCCCTTG	GGGGGCCTCC
	ATCTCCCTTG	GGCCGGGGGC

3.2 Secondary Structure Information

If one or more infiles contain a secondary structure string which consists only of dots ('.') and brackets ('(' and ')'), FASconCAT-G will print additional structure string information to an extra output file named FcC_structure.txt. If sequence concatenation has been chosen via default or "-o -o" command, FASconCAT-G will print basal secondary structure information of the concatenated supermatrix string to the common info file as well (see Table 10). Table 16 lists all additional structure info printed to FcC_structure.txt. Note: only one structure string is allowed per infile and the corresponding string name must be identical among each infile (mind your cases). Otherwise, FASconCAT-G will abort with an error prompt.

Table 16: Additional structure string information printed to the FcC_structure.txt outputfile

Default Information	Supermatrix File	Converted Infiles
Percent & Total Number of Loop Character States	yes	yes
Percent & Total Number of Stem Character States	yes	yes
List of Loop Positions	yes	yes
List of Stem Pairing Positions	yes	yes
List of Sorted Stem Positions	yes	yes

3.3 Hierarchical Order of File Processing Options

To avoid errors during single file processing steps, for example an exclusion of 3rd nucleotide site positions before sequence translation to amino acid character states, FASconCAT-G contains a hierarchical order of single file processing steps:

1. Read IN of infiles and file checking
2. Sequence renaming

3. Sequence translation (nucleotide sequences to amino acid sequences or visce versa)
4. Generation of consensus sequences of predefined sequence blocks
5. RY coding of nucleotide sequences
6. Exclusion of each 3rd nucleotide site position
7. Sequence concatenation
8. Print out

The process order of FASconCAT-G allows for a wide range of optional process combinations. However, some process chains are not possible in a single process run. For instance, it is not possible to RY code nucleotide sequences before translating them to amino acid sequences or to build consensus sequences before the sequence translation process. For tasks of this nature, FASconCAT-G has to be run twice. However, we hope the current process order of FASconCAT-G is useful for most phylogenetic and population genetic studies. If not, please write to the FASconCAT-G help desk (see 3.4).

3.4 License/Help-Desk/Citation

FASconCAT-G v1.0 is written/developed in Perl by Patrick Kück in 2014. It is implemented in Perl and a free software. It can be distributed and/or modified under the terms of the GNU General Public License as published by the Free Software Foundation; either 2 of the license, or (at your option) any later version.
This program is distributed in the hope that it will be useful, but WITHOUT ANY WARRANTY; without even the implied warranty of MERCHANTABILITY or FITNESS FOR A PARTICULAR PURPOSE. See the GNU General Public License for more details.
You should have received a copy of the GNU General Public License along with this program; if not, write to the Free Software Foundation, Inc., 675 Mass Ave, Cambridge, MA 02139, USA.
If you have any problems, error-reports or other questions about FASconCAT-G feel free and write an email to patrick_kueck@web.de which is the official help desk email account for the software. For further free downloadable programs from our institute visit:
http://software.zfmk.de.

If you use FASconCAT-G please cite:
Kück P., Meusemann K. (2010): FASconCAT: Convenient handling of data matrices. Mol. Phylogen. Evol. 56:1115-1118

4 Example Usage

The following examples should show that FASconCAT-G is a suitable and user-friendly tool for complex phylogenetic and population genetic data processing. All different options of FASconCAT-G are combinable.

4.1 Example 1

The following steps can be performed by FASconCAT-G in a single process run:

1. Read IN of two different nucleotide alignment formats (PHYLIP (strict) & FASTA)
2. Generation of strict consensus sequences of predefined sequence blocks in given infiles (-c -c -c)
3. RY coding of remaining sequence states (-b)
4. Exclusion of each third sequence position of given infile sequences (-d)
5. Print OUT of converted infiles and the concatenated supermatrix in PHYLIP (relaxed) format (-o -o -p -p -a)

- COMMAND: -o -o -p -p -a -c -c -c -b -d -s

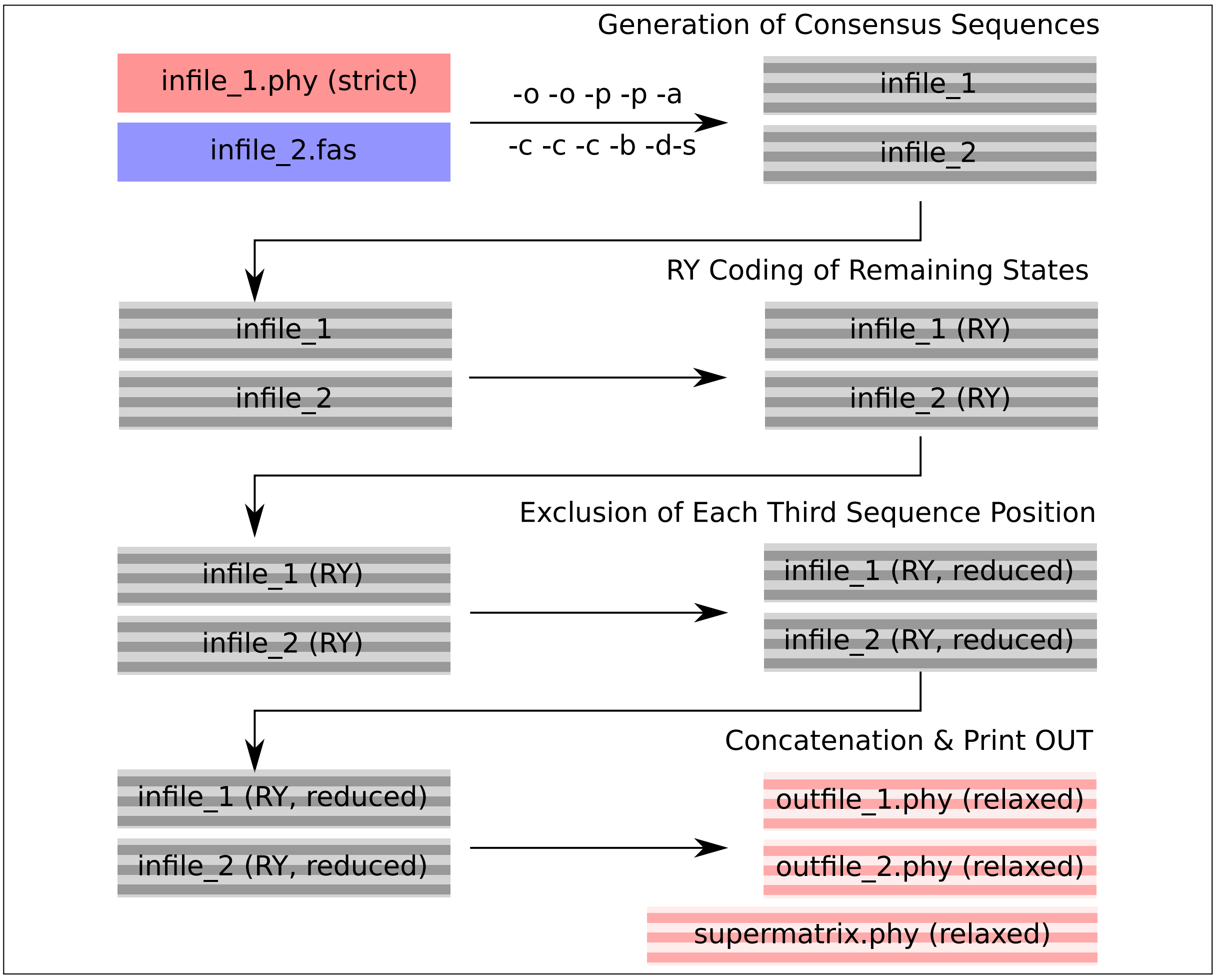

Figure 19: Example usage 1 of combined FASconCAT-G options.

4.2 Example 2

The following steps can be performed by FASconCAT-G in a single process run:

1. Read IN of one PHYLIP (relaxed) formatted nucleotide alignment infile and one FASTA formatted amino acid alignment infile
2. Translation of the amino acid sequences to nucleotide sequences (-e -e)
3. Generation of majority rule consensus sequences of predefined sequence blocks in given infiles (-c -c)
4. Removing of each third sequence position (-d)
5. Print OUT of converted infiles and the concatenated supermatrix in NEXUS (MrBayes) format (-o -o -n -n -a)
6. Print OUT of remaining parsimony informative sites as extra outfiles (-j)

- COMMAND: -o -o -n -n -a -c -c -d -e -e -j -s

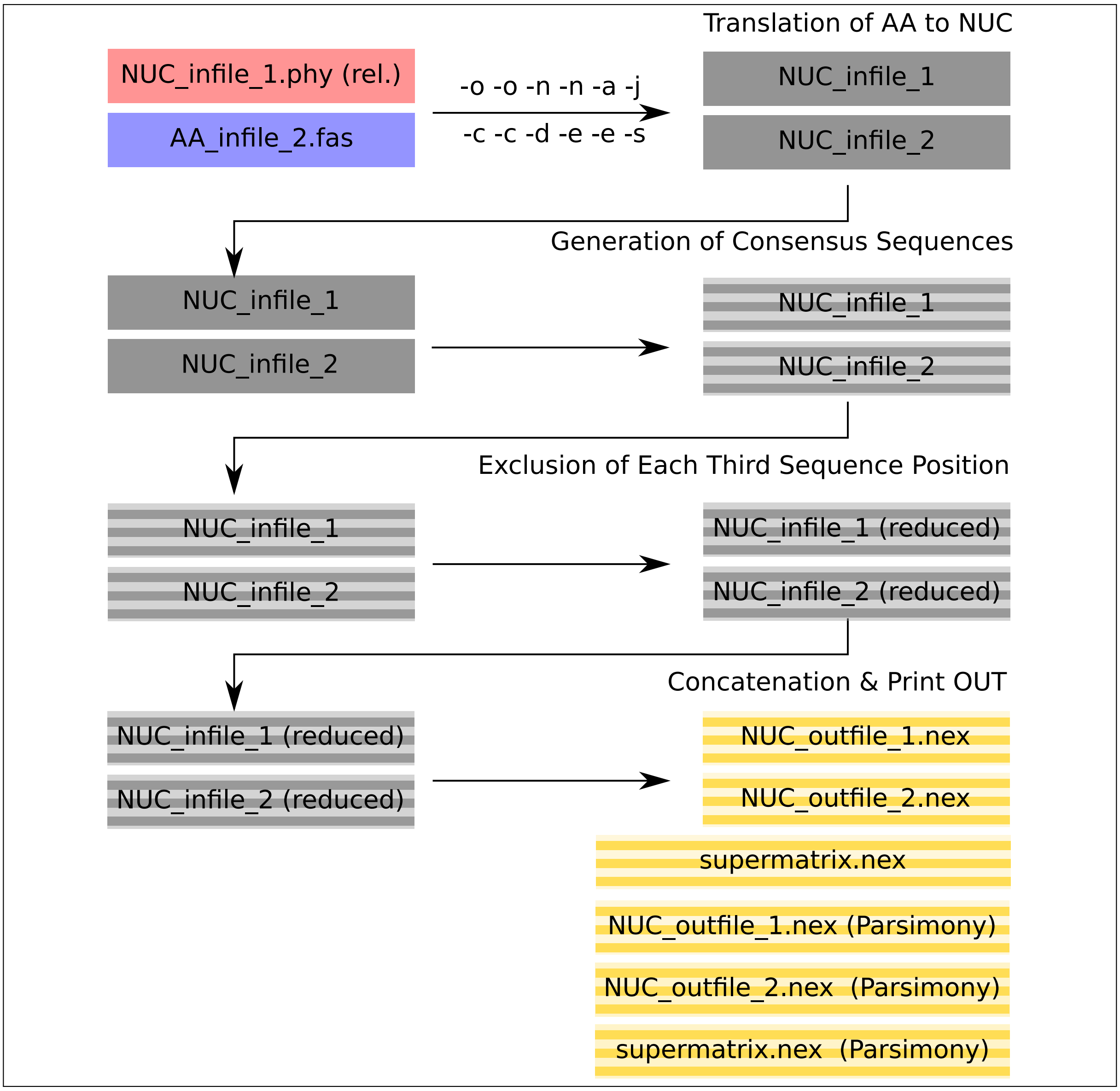

Figure 20: Example usage 2 of combined FASconCAT-G options.

4.3 Example 3

The following steps can be performed by FASconCAT-G in a single process run:

1. Read IN of one PHYLIP (relaxed) formatted nucleotide alignment infile and one FASTA formatted amino acid alignment infile
2. Translation of the nucleotide sequences to amino acid sequences (-e)
3. Generation of most frequent consensus sequences of predefined sequence blocks in given infiles (-c)
4. Print OUT of converted infiles in FASTA and PHYLIP (strict) format (-o -p)

- COMMAND: -o -p -c -e -s

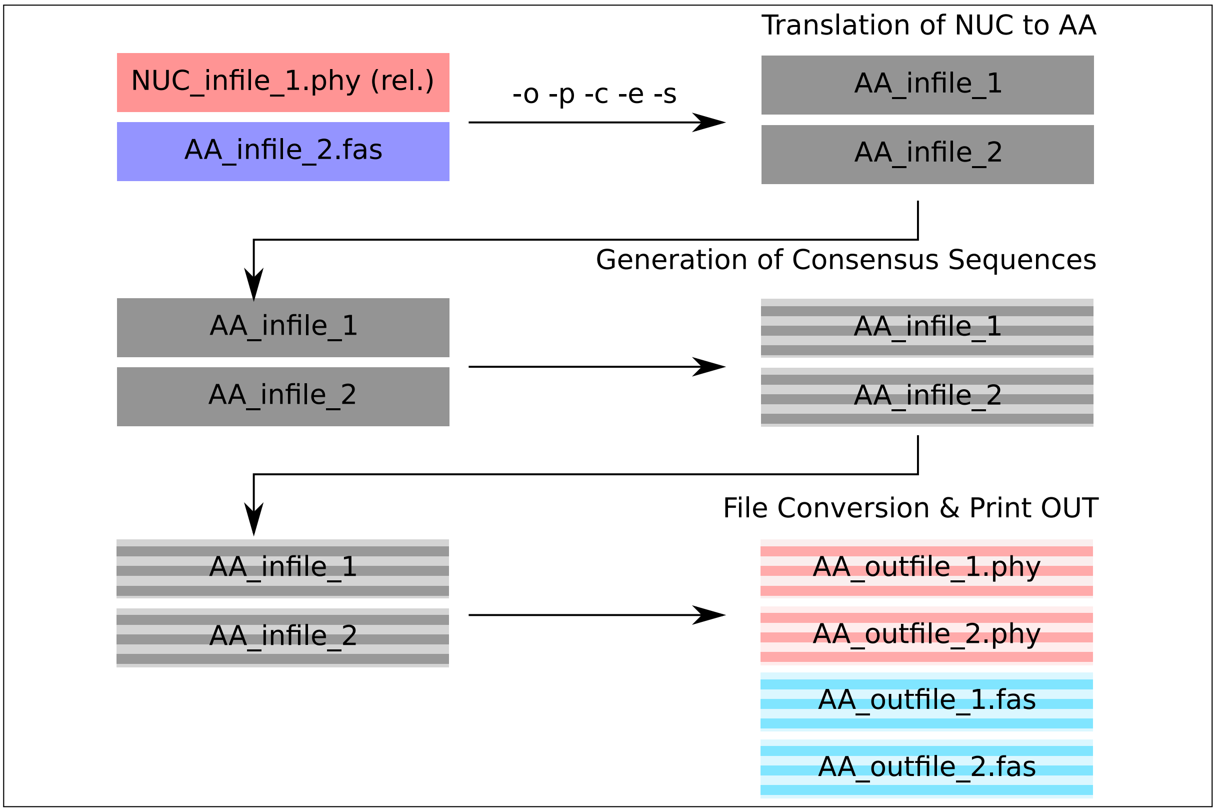

Figure 21: Example usage 3 of combined FASconCAT-G options.

5 Copyright

© by Patrick Kück & Gary C. Longo, June 2014
